# The functional impact of middle Eastern political conflict on mental health and coping behaviors among Egyptian citizens: cross sectional study

**DOI:** 10.1186/s13031-025-00726-5

**Published:** 2025-11-27

**Authors:** Reham Salah Amin Radwan, Esalm B. Ahmed Behery Elazab, Shaimaa A. Senosy, Zahraa M. Mostafa

**Affiliations:** 1 Department of Family Medicine, Faculty of Medicine, Beni-Suef University, Beni-Suef, Egypt; 2 Department of Public Health and Community Medicine, Faculty of Medicine, Beni-Suef University, Beni-Suef, Egypt

**Keywords:** Mental health, Political conflict, Perceived stress, Resilience, Non-displaced civilians

## Abstract

**Background:**

Prolonged political conflict in the Eastern Mediterranean Region has led to widespread instability, with mental health concerns extending outside directly affected populations.

**Aim:**

This study investigates the psychological impact of regional conflict on non-displaced civilians in Egypt, a conflict-neighboring country.

**Methods:**

A cross-sectional study was carried out involving 394 Egyptian adults utilizing validated Arabic versions of the PHQ-9 (depression), GAD-7 (anxiety), PSS-10 (perceived stress), and BRCS (resilient coping). Participants also responded to a single-item measure evaluating the extent to which political conflict interrupted their daily functioning. Data was analyzed using descriptive statistics and comparisons across conflict-impact categories.

**Results:**

Respondents reporting greater functional disruption from regional conflict displayed significantly higher depression, anxiety, and stress scores (*p* < 0.01; η² = 0.13–0.17) and lower resilient coping (*p* < 0.05; η² = 0.02). The highest burden appeared among participants aged 18–25 and those reporting “extreme difficulty.” Females and frequent social media users exhibited greater psychological distress. Non-overlapping 95% CIs supported the robustness and clinical relevance of group differences.

**Conclusions:**

The findings highlight civilians’ psychological vulnerability in politically unstable yet non-war contexts. Indirect conflict exposure, through media, cultural ties, or regional proximity, adversely affects mental health, underscoring the need for targeted screening and resilience-building programs, particularly for youth and digitally active groups.

## Introduction

‏ Internationally, political disputes have reached new peaks in recent years, second only to the surge observed in the early 1990s. The Eastern Mediterranean Region (EMR), a major source of global refugee migration, continues to face multiple violent conflicts. According to the World Health Organization (WHO), the EMR comprises 21 countries, including Afghanistan, Egypt, Iraq, Jordan, Lebanon, Libya, Morocco, Pakistan, Sudan, Syria, Tunisia, and the West Bank and Gaza Strip [1]. Beyond displacement, persistent challenges such as stigma, limited-service capacity, and uneven infrastructure intensify the psychological toll of conflict [2].

Both the frequency and diversity of political disputes have increased. Both at the national level and among the impacted subnational populations or territories, these lower-intensity conflicts can nevertheless have a significant influence on development and human well-being. An expanding body of research that employs micro-level event data to investigate the population-level effects of political conflict on civilians has been driven by the evolving character of political conflict in the twenty-first century as well as the increased availability and quality of datasets that quantify the occurrence of conflict [3].

Many wars, military invasions, civil wars, prolonged conflicts, and protests have taken place in several Arab countries. Some of these crises persisted for years, while others remain ongoing, with the Israeli Palestinian conflict being the longest-lasting. These events led to the collapse of social institutions, economic instability, mass displacement, and widespread casualties. Unsurprisingly, the mental health of affected populations has been profoundly impacted [4]. In fact, evidence shows that even when populations are not directly displaced, cumulative stressors—such as insecurity, social disruption, and economic uncertainty—can produce long-term psychological consequences [5].

Whatever the reasons, these conflicts have significant short- and long-term impacts on the mental health of those involved as well as the generations that follow. Organized psychiatry plays a significant role in promoting mental health, provides appropriate mental health services, and aids in the recovery process from the detrimental effects of violent conflicts among communities on mental health [6].

Around 450 million individuals worldwide suffer from mental or behavioral illnesses, yet only a small percentage of them receive even the most basic therapy. This indicates that there is a growing global burden of mental disorders and a growing treatment gap. Ageing populations and declining public health and infrastructural services will probably result in an increase in the number of cases of mental disorders. It is well recognized that mental illnesses negatively impact role functioning more than many severe, long-term physical illnesses do [7].

The prevalence or severity of untreated mental illnesses are not fully known, particularly in developing nations like Egypt. Egypt’s health care system continues to suffer with numerous issues. Staff and facilities are typically dispersed unevenly, concentrating mostly in cities like Alexandria and Cairo. Compared to international standards, there are substantially fewer mental health specialists [8]. The fact that the services are hospital-based rather than community-based, however, is their biggest challenge. One of the most common issues in Arab nations is the lack of information regarding statistics related to mental health. Community surveys in the field of psychiatry are scarce in Egypt [9]. We require such information for the purpose of planning, developing, training, and integrating mental health into primary healthcare in the future [7].

The difficult burden of comprehending and applying the biopsychosocial model to all three of its domains falls on family doctors. Patients may arrive with a complicated mix of psychological and physical issues. Ideally, medical professionals realize the significance of psychological and social elements and integrate them into the treatment plans for their patients [10].

Several studies confirm the fact that emotional issues are common among patients who see family doctors. Owing to the present managed care environment, which discourages mental health referrals, few of these individuals receive recommendations to psychiatric care. For a variety of reasons, people frequently refuse to see mental health professionals even after being referred. Family doctors should therefore devise plans for managing these individuals while also attending to their medical issues. Specifically, they want an efficient way to include psychotherapy in the patient visit [7].

### Aim of the work

This study aims to assess the association between perceived functional impact of Middle Eastern political conflict and mental health outcomes—specifically depression, anxiety, perceived stress, and coping capacity—among Egyptian civilians.

### Subjects and methods

#### Study design

Cross sectional study.

#### Setting

This study was conducted at Elsabaa Banat Primary Health Care Center, Ismailia Governorate, between May and December 2024. The center was purposively selected for the following reasons.

- Ismailia lies on the west bank of the Suez Canal, bordering the Sinai Peninsula, and has a historical background of displacement and war exposure.
- Elsabaa Banat PHC is centrally located and serves a large and socioeconomically diverse population, making it suitable for recruiting adult participants for questionnaire-based studies.

#### Target population

The study included Egyptian adults aged 18 years or older, born to Egyptian parents. Participants were excluded if they were under 18 years of age, had any known psychiatric, neurological, or chronic medical condition, or intellectual disability, were non-Egyptian nationals, or were members of the police or armed forces. These criteria were applied to focus on psychologically healthy civilian adults and to minimize confounding from pre-existing illness or occupational stressors.

#### Sampling and recruitment

According to a 2021 governmental survey, Ismailia Governorate has 68 primary health care facilities [11]. Elsabaa Banat PHC was purposively selected for its high patient volume, central location, and logistical feasibility. Participants were recruited in person between May and December 2024 using consecutive sampling. Eligible adults attending the center were invited to participate, provided consent, and completed the self-administered questionnaire on site. Of 560 distributed forms, 394 were fully completed and included, yielding a response rate of approximately 70.4%. Questionnaires with more than 10% missing responses were excluded from analysis. No data imputation was performed, and analyses were conducted on complete cases only. No online recruitment was used.

#### Sample size

- Sample size was calculated based on the formula:
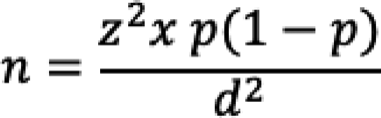
  *n* is the calculated sample size.
  *z* is the statistic that defines the level of confidence required.
  *p* is an estimate of the key indicator to be measured by the survey in the population group of interest, for example, the prevalence of iron deficiency among WRA, expressed as a proportion of that population.
  *d* is the desired level of precision, or the margin of relative error to be obtained.
- For the current study: z = 1.96, (95% confidence level). The expected estimate of the key indicator (*p*) is not known, so we used the value of 0.5 (or 50%) to produce the largest sample size. The value of *d* was set as ± 5%.
- According to this calculation a minimal total hypothesized sample size of 383 adult non-clinical civilian according to the overmentioned inclusion and exclusion criteria was calculated at a two-sided confidence level (1-alpha) 95%, Power (% chance of detecting) 80%, taking into consideration 5% level of significance and 5% precision using Z- test.

#### Study tools

1. **Patient Health Questionnaire-9 (PHQ-9)**

The **Patient Health Questionnaire-9 (PHQ-9)** is a brief, validated tool developed by ***Spitzer et al.*** to screen depression based on DSM-IV criteria. It includes nine items scored from 0 (not at all) to 3 (nearly every day), with total scores ranging from 0 to 27. Severity is categorized as minimal (0–4), mild (5–9), moderate (10–14), moderately severe (15–19), or severe (20–27). The PHQ-9 shows strong internal consistency (Cronbach’s α >0.80). The Arabic version, validated by Sawaya et al. (2016), also demonstrated good reliability (α = 0.857). It can be self- or interviewer-administered in about 2–5 min, Arabic versions used the same validated cutoffs as the original English instruments [12].

2. **Generalized Anxiety Disorder Scale-7 (GAD-7)**

The **Generalized Anxiety Disorder Scale-7 (GAD-7)**, developed by ***Spitzer et al.***, is a quick and reliable tool for screening anxiety symptoms. It includes seven items scored from 0 (not at all) to 3 (nearly every day), with total scores ranging from 0 to 21, categorized as minimal (0–4), mild (5–9), moderate (10–14), or severe (15–21). It demonstrates excellent reliability (Cronbach’s α = 0.92) and validity. The Arabic version, validated by Sawaya et al. (2016), also shows solid psychometric strength (α = 0.763). It can be completed in about 2–5 min, making it suitable for self- or interviewer administration, Arabic versions used the same validated cutoffs as the original English instruments [13].

3. **Perceived Stress Scale (PSS-10)**

The **Perceived Stress Scale (PSS-10)**, developed by ***Cohen et al.*** assesses how stressful individuals perceive their lives over the past month. It consists of 10 items rated on a 5-point scale from 0 (never) to 4 (very often), yielding a total score between 0 and 40. Scores are interpreted as low (0–13), moderate (14–26), or high (27–40) stress. The PSS-10 has demonstrated strong reliability (Cronbach’s α = 0.78–0.91) across cultures. The Arabic version, validated by ***Almadi et al.***, also showed good internal consistency (α = 0.80). It takes about 4–5 min to complete and is suitable for self- or interviewer administration, Arabic versions used the same validated cutoffs as the original English instruments [14, 15].

4. **Brief Resilient Coping Scale (BRCS)**

The Brief Resilient Coping Scale (BRCS), developed by ***Sinclair and Wallston***, is a concise tool designed to assess how effectively individuals cope with stress in adaptive ways. It includes 4 items rated on a 5-point scale from 1 (does not describe me at all) to 5 (describes me very well), with total scores ranging from 4 to 20. Scores classify coping as low (4–13), moderate (14–16), or high (17–20) resilience. The original scale showed acceptable internal consistency (α = 0.69), and the Arabic version—validated in an Egyptian context by ***Khalaf and Al-Said*** demonstrated strong reliability (α = 0.83) and sound construct validity. It is very quick to complete (in about 2 min), making it ideal for both self-report and interviews, Arabic versions used the same validated cutoffs as the original English instruments [16, 17].

Media exposure and personal ties to conflict zones were considered as contextual influences but were not directly measured in this study.

5. **Perceived Functional Impact of Political Conflict**

Participants were asked: ***[How much have political conflicts in the Middle East made it difficult for you to do your work***,*** study***,*** fulfill your responsibilities at home***,*** or deal with people?]***. Response options were no difficulty, somewhat difficult, very difficult, and extremely difficult. The item was analyzed as a categorical variable to assess perceived functional disruption. Although this was not part of a standardized scale, it was designed to capture context-specific functional impact related to regional conflict. Similar single-item questions have been widely used in public-health and social-epidemiological research to assess perceived burden, stress, or health status when no established scale exists for the construction [18–20].

### Statistical analysis

Data were analyzed using SPSS version 26 (IBM Corp., Armonk, NY, USA). Tests for normality and homogeneity of variance were performed prior to applying *t*-tests and one-way ANOVA for group comparisons. Effect sizes were calculated to indicate the magnitude of differences, using Cohen’s *d* for pairwise analyses and eta squared (η²) for ANOVA, with 95% confidence intervals reported to reflect estimate precision. Given the small cell sizes in the highest exposure categories, results were interpreted cautiously, and sensitivity analyses using merged categories yielded similar directional findings. Multiple comparisons were restricted to primary hypotheses, and exploratory analyses were clearly identified. A multiple linear regression model was conducted to identify predictors of resilience; although the model explained a modest proportion of variance (Adjusted R² = 0.142), variance inflation factor (VIF) values below 3 indicated no problematic multicollinearity.

## Results

1. **Sociodemographic characteristics**

A total of 394 participants were included in the study. The sample was predominantly female (58.4%), with males constituting 41.6%. The majority of respondents (53.8%) were aged between > 34 and ≤ 45 years. Regarding marital status, 64.5% were married, 28.4% were never married, and 7.1% were divorced, separated, or widowed. Educationally, 44.2% held higher education degrees, 32.5% had postgraduate qualifications, and 12.4% had average education levels (Table 1).

2. **Depression severity (PHQ-9)**

**Fig. 1:**
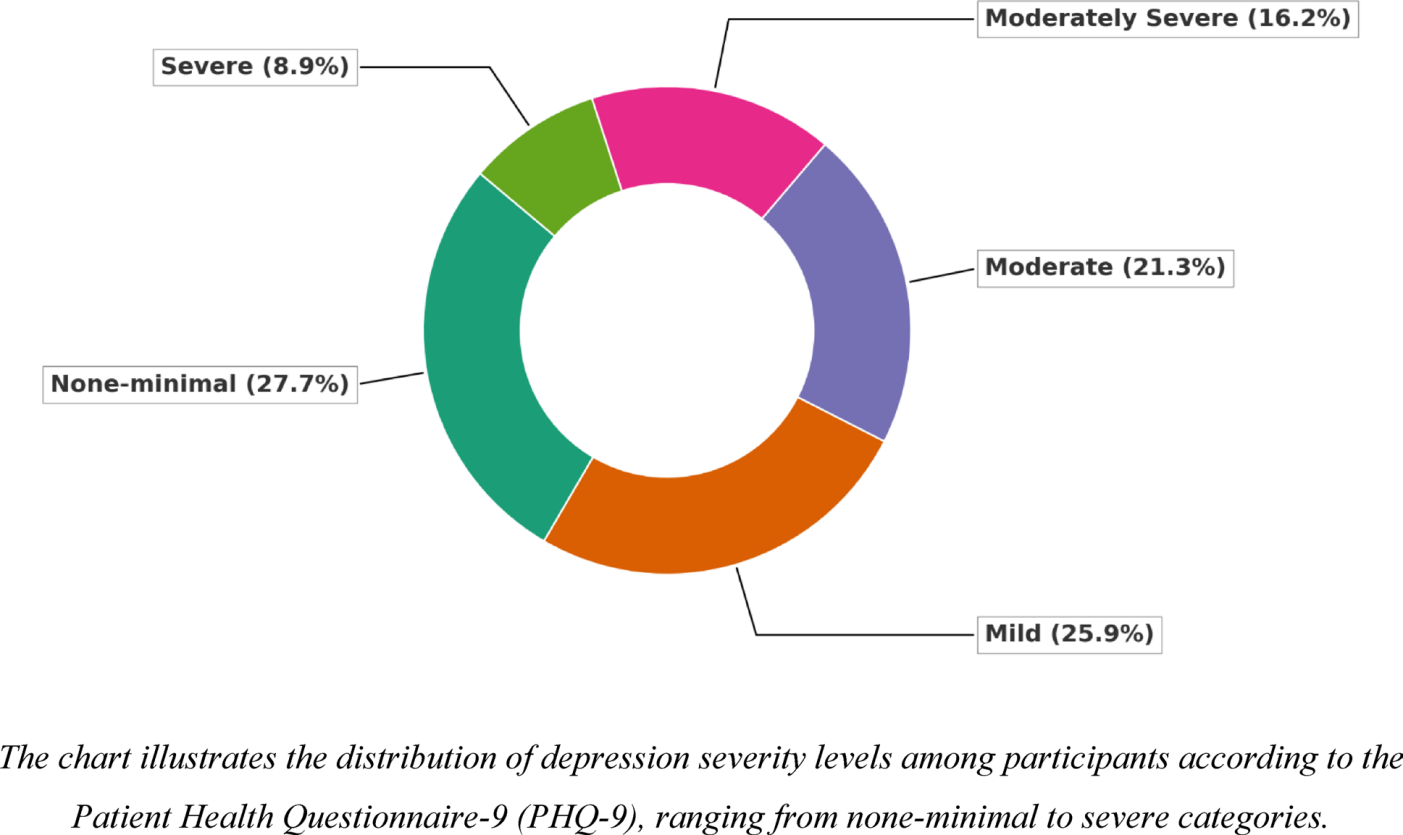
Depression Severity among studied participants by patient health questionnaire −9 (PHQ-9). The chart illustrates the distribution of depression severity levels among participants according to the Patient Health Questionnaire-9 (PHQ-9), ranging from none-minimal to severe categories

Depressive symptoms were reported by 72.3% of participants, with 21.3% experiencing moderate, 16.2% moderately severe, and 8.9% severe depression. Only 27.7% reported minimal or no depressive symptoms. Females had significantly higher mean PHQ-9 scores (11.01 ± 6.57) than males (8.06 ± 5.62), and younger participants, particularly those under 35 years, exhibited greater severity. Depression scores were also elevated in never-married individuals and those with average educational attainment (Fig. 1). As summarized in (Table 2), these differences were statistically significant across gender (*p* < 0.001, η² = 0.052), age (*p* < 0.001, η² = 0.082), marital status (*p* = 0.004, η² = 0.028), and education (*p* = 0.002, η² = 0.036), indicating small-to-moderate practical effects supported by non-overlapping 95% confidence intervals.

3. **Perceived stress (PSS)**

**Fig. 2:**
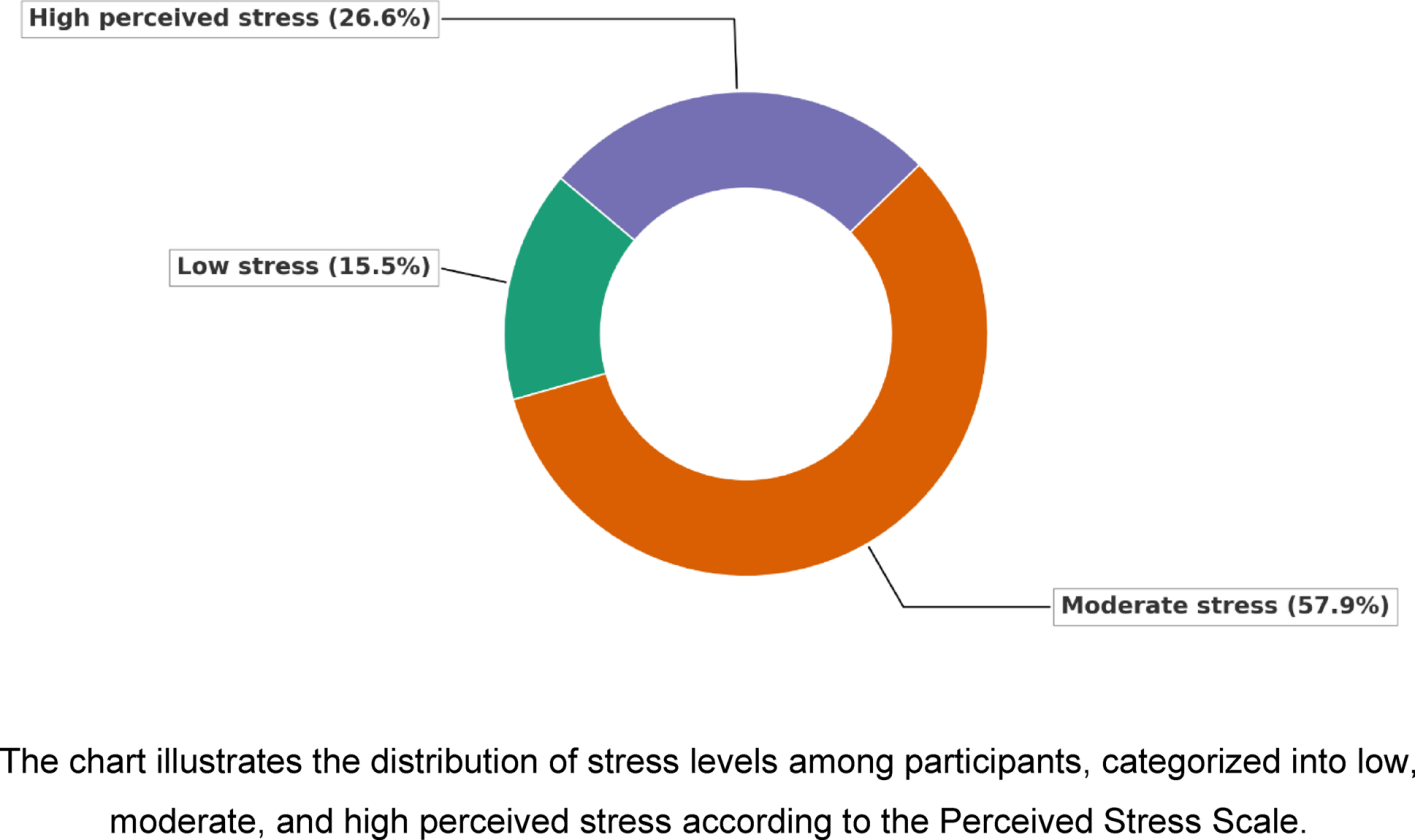
Stress level among studied participants by Perceived Stress Scale (PSS). The chart illustrates the distribution of stress levels among participants, categorized into low, moderate, and high perceived stress according to the Perceived Stress Scale

Most participants (57.9%) reported moderate stress levels, and 26.6% experienced high stress. Mean PSS scores were significantly higher among females (22.73 ± 5.91) than males (18.98 ± 7.73). The highest stress levels were observed in the 25–34 age group (24.69 ± 4.99) and never-married participants (23.28 ± 5.80). Educational level was not significantly associated with stress, though slight variations were noted (Fig. 2). As shown in (Table 2), these associations were significant for gender (*p* < 0.001, η² = 0.071) and age (*p* < 0.001, η² = 0.102), reflecting moderate-to-large effects, while marital and educational differences were comparatively smaller (η² = 0.037 and 0.013, respectively).

4. **Anxiety severity (GAD-7)**

**Fig. 3:**
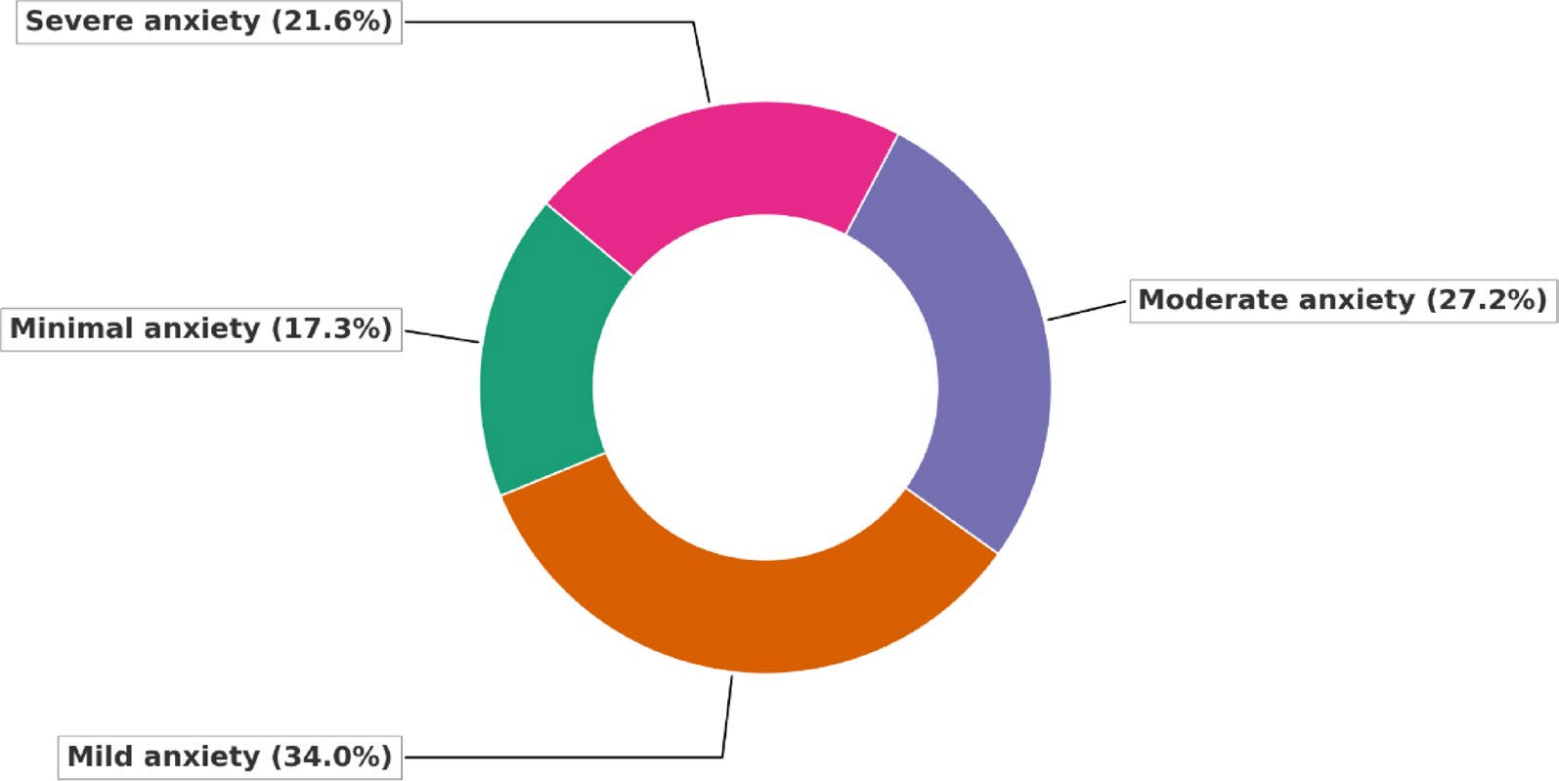
Anxiety Severity among studied participants by generalized anxiety disorder (GAD-7). The chart depicts the distribution of anxiety severity levels among participants, categorized as minimal, mild, moderate, and severe according to the GAD-7 scale

A total of 82.7% of participants reported anxiety symptoms ranging from mild to severe, with 27.2% classified as moderate and 21.6% as severe. Severe anxiety was more common among females (25.7%) than males (15.9%), and most prominent in the 25–34 age group. Participants with average education reported the highest anxiety scores (mean GAD-7 = 12.19 ± 4.68) (Fig. 3). According to (Table 2), significant group differences were observed by gender (*p* < 0.001, η² = 0.034), age (*p* = 0.004, η² = 0.038), and education (*p* = 0.021, η² = 0.026), with the 95% CIs supporting these mean differences. Marital status showed no significant effect (*p* = 0.521).

5. **Resilient coping patterns (BRCS)**

**Fig. 4:**
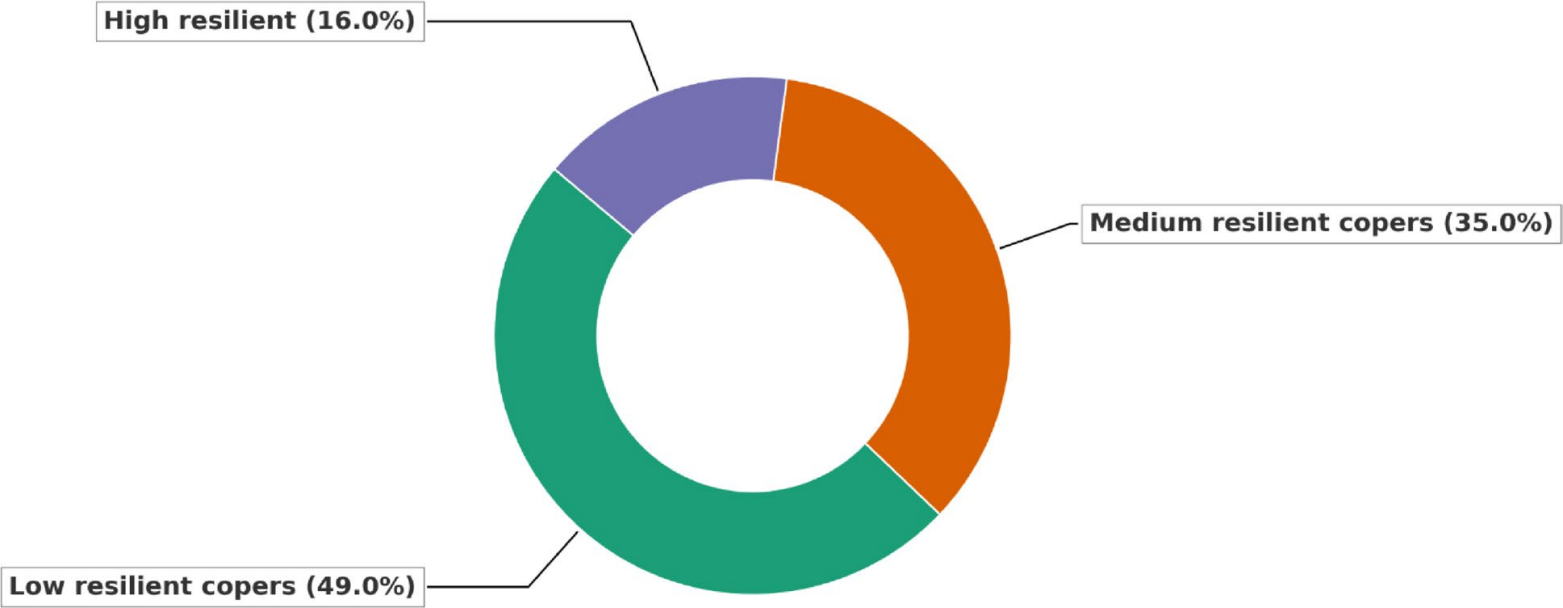
Resilience Level Among Studied Participants by Brief Resilient Coping Scale (BRCS). The chart illustrates the distribution of resilience levels among participants, categorized into low, medium, and high resilient copers according to BRCS classifications

Low resilience was found in 49.0% of the sample, moderate in 35.0%, and high in only 16.0%. Males had significantly higher BRCS scores than females (14.04 ± 2.61 vs. 13.01 ± 3.48, *p* < 0.001). Greater resilience was observed among older participants (> 45 years), those with higher education, and divorced/widowed individuals. Low resilience was notably more prevalent among younger, unmarried, and less-educated participants (Fig. 4). (Table 2) highlights these trends, showing significant differences across gender, age, marital status, and education (*p* < 0.001 for all), with moderate effect sizes (η² = 0.026–0.103). The narrow 95% confidence intervals indicate precise mean estimates, and the direction of change suggests that resilience increases with age, education, and life experience.

6. **Correlation between resilience and psychological distress**

**Fig. 5:**
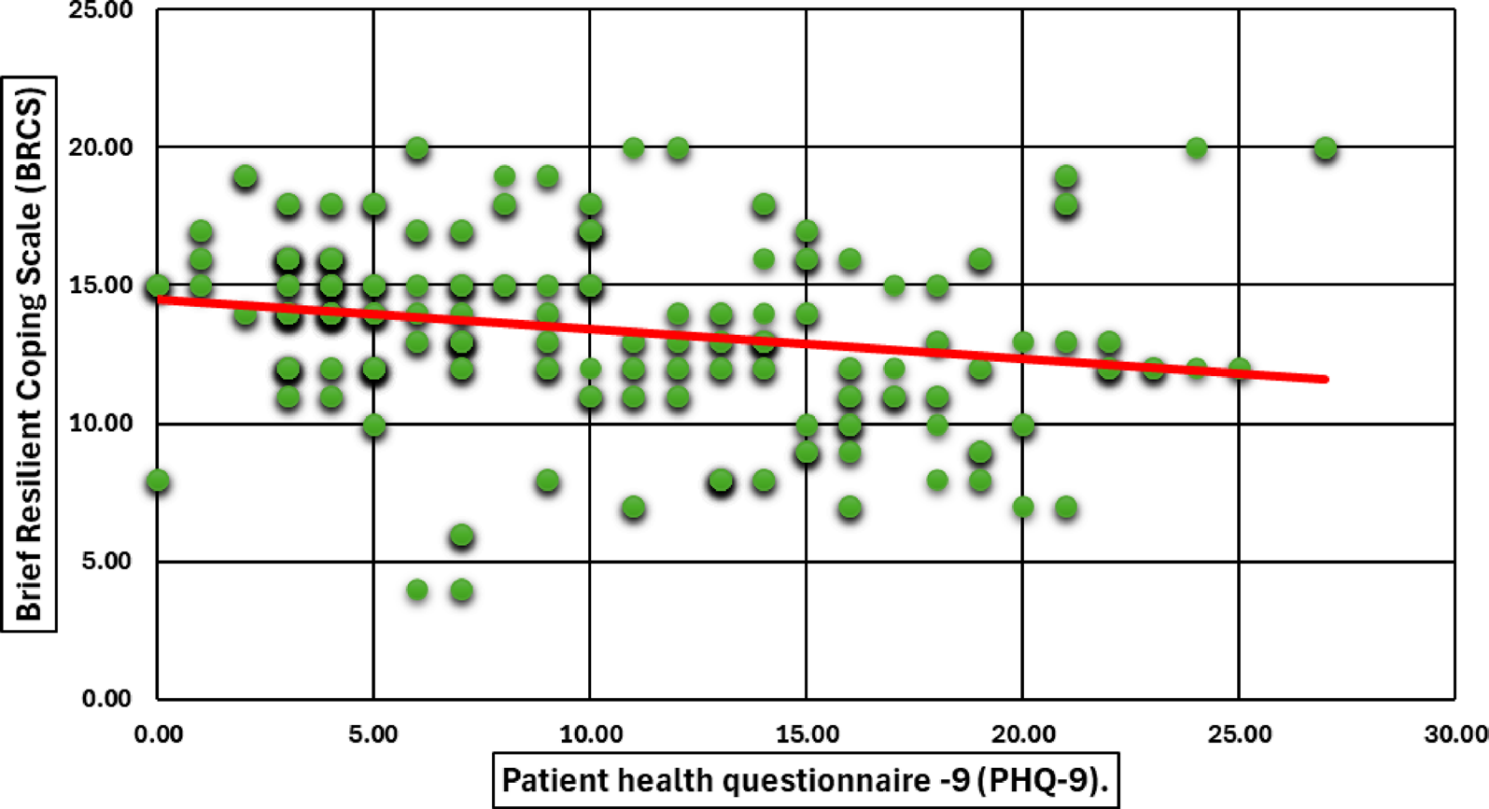
Pearson Correlation Between Coping Behavior (BRCS) and Patient health questionnaire −9 (PHQ-9) Among Participants. A scatter plot depicts the correlation between participants’ coping behavior, measured by the Brief Resilient Coping Scale (BRCS), and depression severity, assessed by the Patient Health Questionnaire-9 (PHQ-9). A negative trend line indicates an inverse association, suggesting that higher depression scores are related to lower resilient coping

**Fig. 6:**
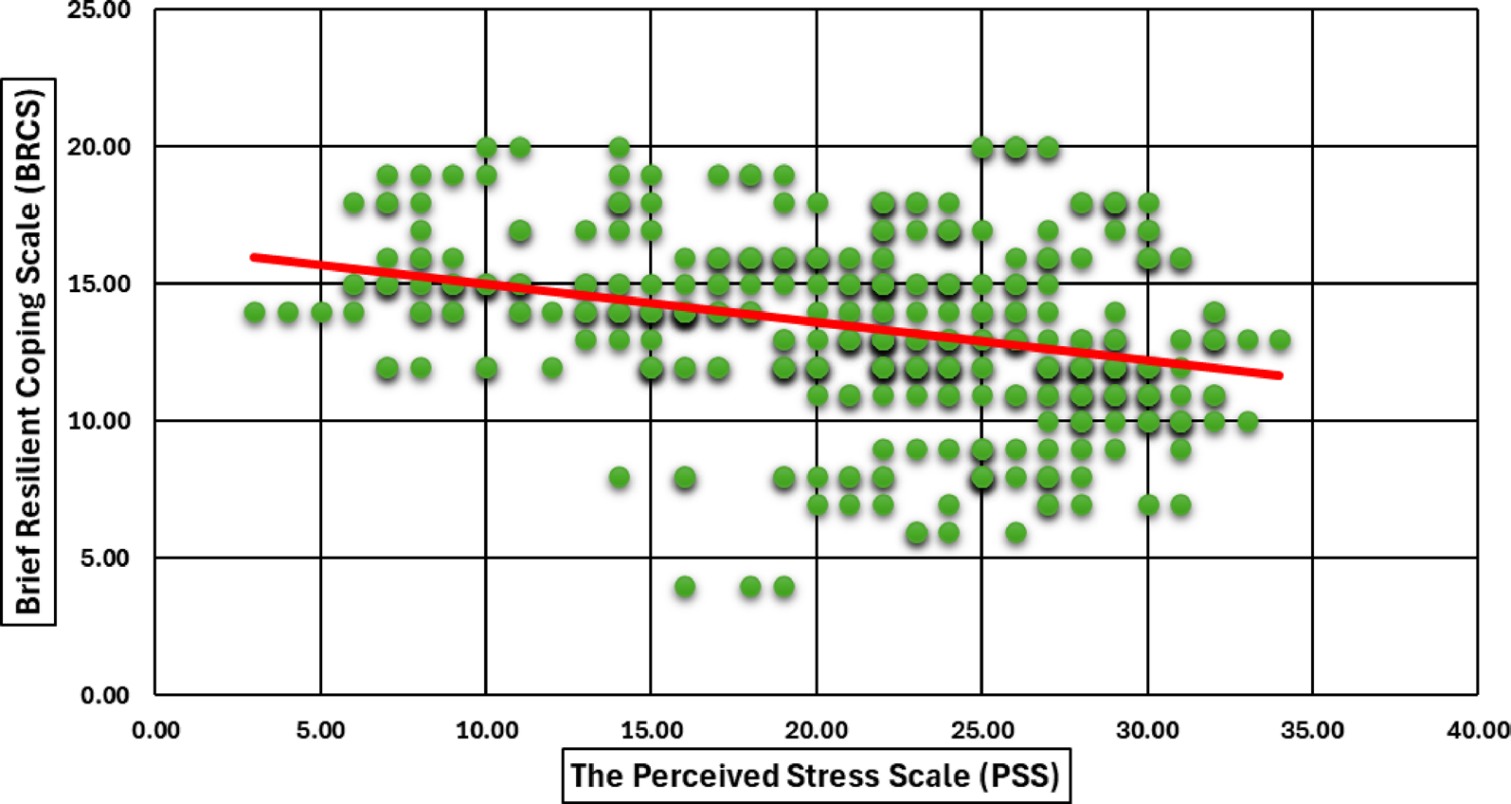
Pearson Correlation Between Coping Behavior (BRCS) and Perceived Stress Scale (PSS) Among Participants. A scatter plot illustrates the correlation between coping behavior, assessed using the Brief Resilient Coping Scale (BRCS), and perceived stress levels measured by the Perceived Stress Scale (PSS). The negative trend line suggests that higher levels of perceived stress are accompanying lower resilient coping capacity

**Fig. 7:**
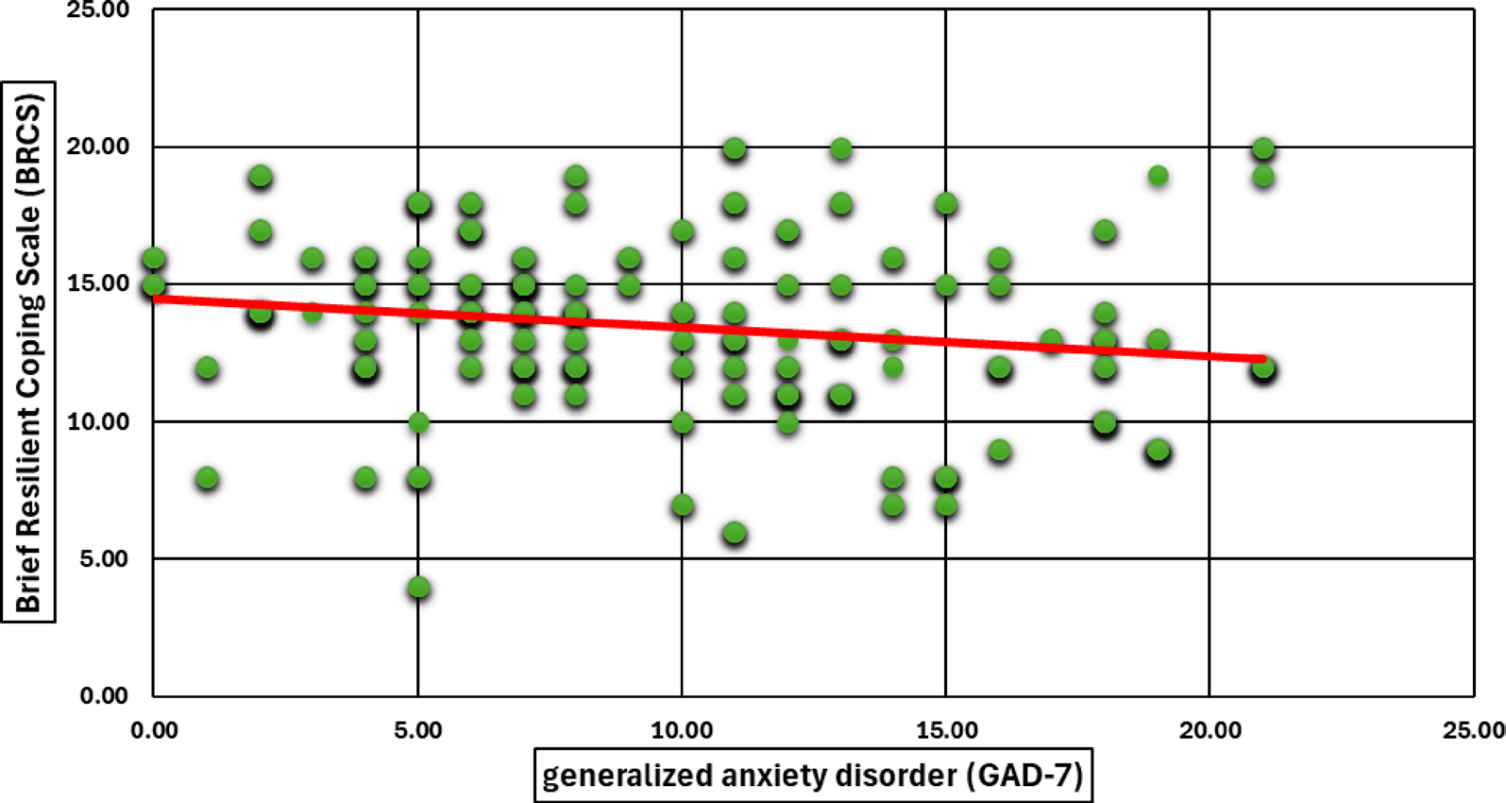
Pearson Correlation Between Coping Behavior (BRCS) and Generalized Anxiety Disorder (GAD-7). A scatter plot demonstrates the correlation between coping behavior, as measured by the Brief Resilient Coping Scale (BRCS), and anxiety levels, assessed using the GAD-7 scale. The downward trend line suggests a negative association, indicating that higher anxiety symptoms may correspond to lower levels of resilient coping

Pearson correlation analysis demonstrated significant negative associations between BRCS scores and mental health measures: PHQ-9 (*r* = − 0.213), PSS (*r* = − 0.306), and GAD-7 (*r* = − 0.178), all *p* < 0.001. These results indicate that greater psychological distress is associated with lower coping capacity (Figs. 5, 6 and 7).

7. **Predictors of resilience (Regression analysis)**

A multiple linear regression model identified age (β = 0.216, *p* < 0.001), education level (β = 0.109, *p* = 0.023), and perceived stress (β = − 0.248, *p* < 0.001) as significant predictors of resilience. The model accounted for 15.7% of the variance in BRCS scores (Adjusted R² = 0.142). Gender, marital status, depression, and anxiety were not independent predictors (Table 3).

8. **Functional impact of political conflict**

**Fig. 8:**
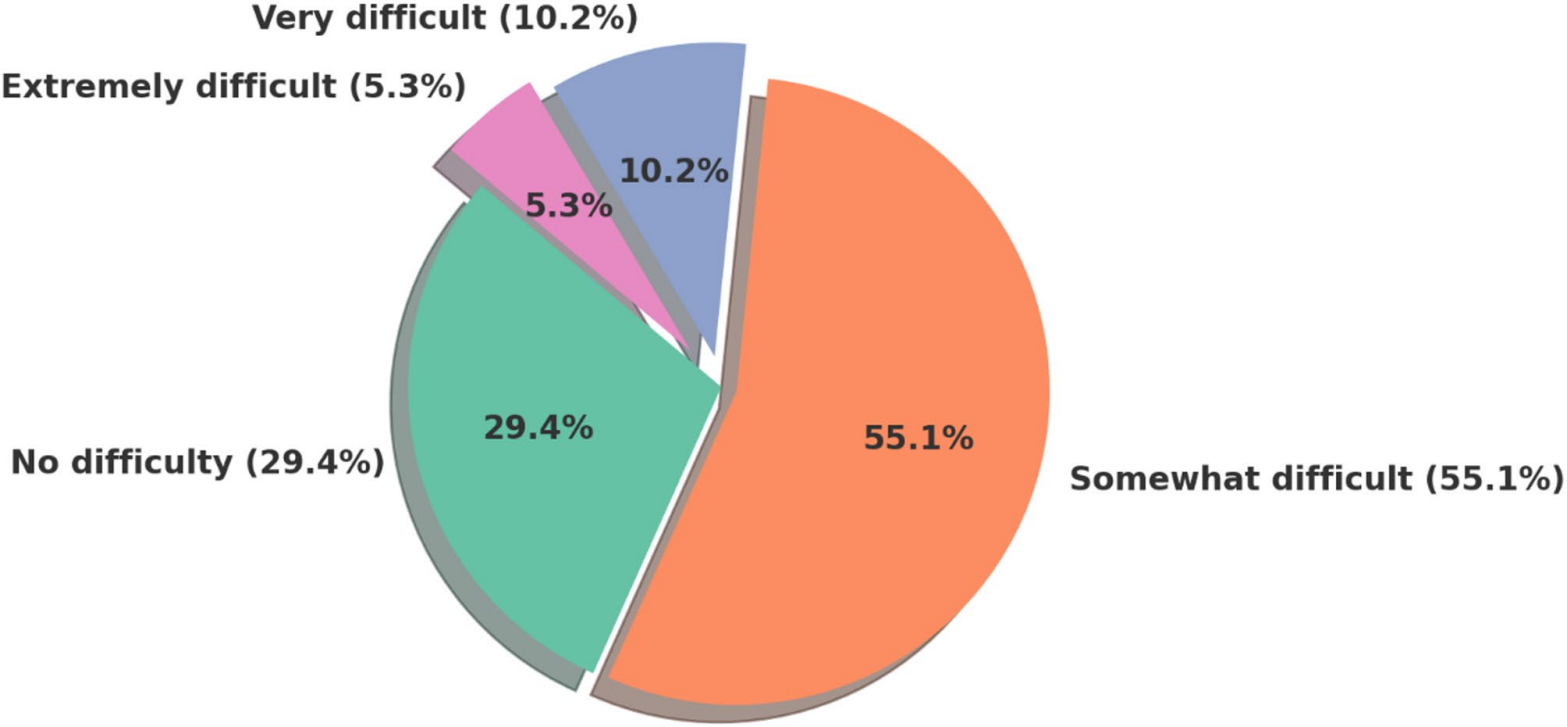
Perceived difficulty in dealing with life aspects due to political conflicts in themiddle east. The pie chart illustrates varying degrees of difficulty among participants, with the majority (55.1%)reporting their experience as “somewhat difficult,” followed by 29.4% indicating “no difficulty.” Smallerproportions described their situation as “very difficult” (10.2%) or “extremely difficult” (5.3%),highlighting that over two-thirds of respondents experienced at least some level of difficulty in coping withthe ongoing political context

Among respondents, 47.6% of those aged 18–25 years reported that regional political conflict made it “extremely difficult” to function in daily life. Perceived disruption was significantly associated with age and marital status, with younger and never-married individuals reporting greater difficulty. While females reported more difficulty than males, the difference was not statistically significant (Fig. 8).

9. **Mental health outcomes by perceived conflict impact**

Participants who perceived political conflict as having an “extremely difficult” impact on their daily functioning confirmed markedly higher mean scores for depression (PHQ-9: 16.48 ± 6.35; 95% CI = 13.59–19.36), stress (PSS: 27.10 ± 4.52; 95% CI = 25.04–29.15), and anxiety (GAD-7: 15.95 ± 4.28; 95% CI = 14.00–17.90), compared with participants who stated “no difficulty.” These differences were statistically significant (*p* < 0.001 for all), with large effect sizes for PHQ-9 (η² = 0.170), PSS (η² = 0.149), and GAD-7 (η² = 0.137), indicating that perceived functional disruption accounted for a substantial portion of the variance in psychological distress. Although resilience (BRCS) also differed significantly across groups (*p* = 0.031; η² = 0.022), the trend was inconsistent—participants reporting greater difficulty showed slightly higher mean scores, suggesting a complex adaptive coping response under high-stress conditions (Table 4).

Reported prevalence estimates reflect symptom screening outcomes rather than diagnostic classifications and should be interpreted within the context of community-based sampling during a period of regional instability.

## Discussion

Armed conflicts have well-documented psychological consequences for directly affected populations; however, less attention has been paid to their impact on non-displaced civilians residing in neighboring regions. This study contributes to the growing body of evidence highlighting the indirect mental health toll of regional conflicts, focusing on Egyptian citizens exposed to the 2023 Gaza War through media, sociopolitical discourse, and cultural solidarity. Indirect exposure—characterized by repeated engagement with distressing news, emotional identification with victims, and national proximity to the conflict—can elicit levels of psychological distress comparable to those experienced in more directly affected populations [21–23].

**Table 1:** Sociodemographic characteristics of the studied participants; (N = 394)

		Frequency	%
Gender	Male	164	41.6
	Female	230	58.4
Age	≥ 18 to ≤ 25 years old	69	17.5
	> 25 to ≤ 34 years old	48	12.2
	> 34 to ≤ 45 years old	212	53.8
	> 45 to ≤ 60 years old	45	11.4
	> 60 years old	20	5.1
Marital status	Married	254	64.5
	Never married	112	28.4
	Divorced/separated/widowed	28	7.1
Educational level	Primary	49	12.4
	Secondary	43	10.9
	University	174	44.2
	Postgraduate Studies	128	32.5

This table illustrates the distribution of participants by gender, age group, marital status, and educational level, showing the demographic composition of the study sample

Our findings demonstrate a substantial mental health burden among civilians in Ismailia Governorate. The majority of participants reported symptoms of depression (72.3%), anxiety (82.7%), and moderate to high stress (84.5%). These rates are markedly elevated, particularly given the non-displaced nature of the sample, and mirror patterns observed in conflict-adjacent communities in Lebanon, Iraq, and Palestine [24–26]. However, they considerably exceed the pooled prevalence estimates reported among civilians indirectly exposed to conflict in global meta-analyses. ***Charlson et al.*** estimated that approximately one in five people in conflict-affected settings experience depression, anxiety, or PTSD, with an overall prevalence of 22.1% [27], while ***Steel et al.*** found a combined prevalence of 30.8% for mental disorders among conflict-exposed but non-displaced populations across 40 countries [28]. This discrepancy may reflect contextual differences, including Ismailia’s border location, historical displacement during past wars, and its population’s strong media engagement and cultural identification with affected groups, all of which may heighten emotional reactivity and perceived stress even in the absence of direct exposure. Psychological distress was significantly associated with sociodemographic variables: younger age, female gender, unmarried status, and lower educational attainment consistently predicted worse outcomes. These results, align with previous research indicating that these groups are more vulnerable to psychological disruption under conditions of chronic stress and vicarious trauma [29, 30]. It is also possible that concurrent economic uncertainty and post-pandemic stressors contributed to elevated distress levels, compounding the psychological burden linked to regional political instability in the current analysis.

Resilience emerged as a critical moderating factor in the psychological response to indirect conflict exposure. Consistent with previous studies, lower resilience was more evident among younger, unmarried, and less-educated individuals, who also exhibited greater levels of stress and depressive symptoms [25, 31]. This pattern supports the notion that resilience acts as a protective psychological resource yet is eroded by sustained emotional strain and sociopolitical instability. The finding that some individuals maintained adaptive coping despite high perceived disruption suggests the presence of culturally embedded resilience mechanisms—such as social solidarity, religious coping, and collective identity—that may buffer the psychological impact of regional conflict [26, 32]. These observations reinforce the need for community-based resilience-building interventions that strengthen coping resources among youth and socioeconomically disadvantaged groups in conflict-adjacent settings.

**Table 2:** Mean scores and statistical differences in depression (PHQ-9), Stress (PSS), anxiety (GAD-7), and resilience (BRCS) by sociodemographic characteristics (N = 394)

	(PHQ-9) Score				(PSS) Score				(GAD-7) Score				(BRCS) Score			
	Mean ± SD	95% CI	*p*-value	**(η²)**	Mean ± SD	95% CI	*p*-value	**(η²)**	Mean ± SD	95% CI	*p*-value	**(η²)**	Mean ± SD	95% CI	*p*-value	**(η²)**
**Gender**																
Male (n = 164)	8.06 ± 5.62	7.19–8.93	< 0.001*	**0.052 (M)**	18.98 ± 7.73	17.79–20.17	< 0.001*	**0.071 (M)**	8.59 ± 5.48	7.75–9.44	< 0.001*	**0.034** **(S–M)**	14.04 ± 2.61	13.64–14.45	0.001*	**0.026** **(S–M)**
Female (n = 230)	11.01 ± 6.57	10.16–11.86			22.73 ± 5.91	21.97–23.50			10.59 ± 5.17	9.92–11.26			13.01 ± 3.48	12.56–13.46		
**Age**																
18–25 yrs (n = 69)	12.26 ± 6.31	10.75–13.78	< 0.001*	**0.082 (M)**	22.68 ± 5.96	21.25–24.11	< 0.001*	**0.102 (M–L)**	10.45 ± 5.50	9.13–11.77	0.004*	**0.038** **(S–M)**	12.41 ± 3.81	11.49–13.32	< 0.001*	**0.103 (M–L)**
> 25–34 yrs (n = 48)	12.58 ± 4.98	11.14–14.03			24.69 ± 4.99	23.24–26.14			12.19 ± 4.68	10.83–13.55			12.25 ± 3.58	11.21–13.29		
> 34–45 yrs (n = 212)	9.07 ± 6.46	8.20–9.94			21.20 ± 7.09	20.24–22.16			9.08 ± 5.37	8.36–9.81			13.43 ± 2.83	13.05–13.82		
> 45–60 yrs (n = 45)	7.78 ± 4.95	6.29–9.27			17.02 ± 6.96	14.93–19.11			9.09 ± 4.65	7.69–10.48			15.24 ± 2.39	14.53–15.96		
> 60 yrs (n = 20)	6.55 ± 6.59	3.47–9.63			16.60 ± 6.57	13.52–19.68			10.20 ± 6.70	7.06–13.34			15.90 ± 1.41	15.24–16.56		
**Marital Status**																
Married (n = 254)	9.29 ± 6.27	8.51–10.06	0.004*	**0.028 (S–M)**	20.41 ± 7.09	19.53–21.29	0.001*	**0.037 (S–M)**	9.78 ± 5.48	9.11–10.46	**0.521**	**0.003 (Negligible)**	13.81 ± 2.91	13.45–14.17	< 0.001*	**0.081 (M)**
Never Married (n = 112)	11.38 ± 6.22	10.21–12.54			23.28 ± 5.80	22.19–24.36			9.97 ± 5.12	9.02–10.93			12.14 ± 3.55	11.48–12.81		
Divorced/Separated/Widowed (n = 28)	7.89 ± 6.59	5.34–10.45			19.68 ± 8.41	16.42–22.94			8.68 ± 5.68	6.48–10.88			15.32 ± 2.16	14.48–16.16		
**Educational Level**																
Primary (n = 49)	12.45 ± 7.54	10.28–14.61	0.002*	**0.036 (S–M)**	22.00 ± 5.71	20.36–23.64	**0.161**	**0.013 (Small)**	11.63 ± 6.65	9.72–13.54	0.021*	**0.026 (S–M)**	12.49 ± 2.79	11.69–13.29	0.001*	**0.042 (M)**
Secondary (n = 174)	9.57 ± 6.03	8.67–10.47			21.07 ± 7.60	19.94–22.21			9.93 ± 5.33	9.13–10.72			13.14 ± 3.34	12.64–13.64		
University (n = 128)	8.66 ± 6.29	7.56–9.76			20.39 ± 6.42	19.27–21.51			8.87 ± 4.90	8.01–9.72			14.34 ± 2.79	13.85–14.82		
Postgraduate (n = 43)	10.95 ± 5.47	9.27–12.64			22.95 ± 6.99	20.80–25.10			9.60 ± 4.98	8.07–11.14			13.09 ± 3.52	12.01–14.18		

This table illustrates mean ± standard deviation (SD), 95% confidence intervals (CIs), p-values, and effect sizes (η²) for PHQ-9, PSS, GAD-7, and BRCS by sociodemographic characteristics. Gender differences were tested using the independent samples t-test, while age, marital status, and education were analyzed by one-way ANOVA with LSD post-hoc tests. A p-value < 0.05 was considered significant*. The 95% CI reflects the range of the true population mean, and effect size (η²) indicates the proportion of variance explained: small (0.01–0.05), moderate (0.06–0.13), and large (≥ 0.14). Labels (S–M), (M), (M–L), and (Negligible) denote the observed magnitude of effect

The sample predominantly comprised middle-aged, educated Egyptian adults, with a higher representation of females and married individuals. These characteristics are relevant given well-established associations between age, gender, marital status, and vulnerability to psychological distress. Similar female-skewed participation has been reported in regional studies investigating war-related mental health impacts, such as ***Hendawy et al.***, where two-thirds of university students surveyed were female, and ***Najem et al.***, who observed comparable gender distributions among Lebanese adults during the Gaza conflict [24, 33]. Although younger populations often dominate war-related mental health research, the current study provides insight into a more mature civilian demographic with family and occupational responsibilities—factors that may heighten perceived stress and anxiety under conditions of regional instability. The predominance of female participants may also reflect greater willingness to disclose emotional distress, consistent with gender-based differences in help-seeking and affective reactivity documented in prior literature. Collectively, these demographic trends emphasize the complex interplay of gender, age, and sociocultural roles in shaping psychological responses to indirect conflict exposure.

Comparable findings have been documented both regionally and globally. ***Najem et al.***, reported elevated anxiety among Lebanese adults indirectly exposed to the 2023 Gaza conflict, largely driven by media engagement and intolerance of uncertainty [24]. Similarly, ***Hendawy et al.*** identified social media exposure as a key predictor of psychological distress among Egyptian and Jordanian students [33]. Qualitative studies from Gaza further highlight that chronic uncertainty, restricted mobility, and repeated exposure to threat sustain anxiety even among mental health professionals [23]. Collectively, these findings reinforce that indirect exposure to political violence can evoke sustained anxiety and functional impairment, particularly among younger adults, women, and those with fewer educational or socioeconomic buffers.

Beyond anxiety, resilience emerged as a critical determinant of psychological adaptation. Consistent with regional and international evidence, lower resilience was most evident among younger, unmarried, and less-educated individuals—groups simultaneously burdened with higher emotional distress [25, 31]. ***Hendawy et al.*** and ***Najem et al.*** also found that emotional identification with conflict and excessive social media engagement weaken coping mechanisms by magnifying perceived threat [24, 33]. Conversely, ***Qamar*** and ***Diab et al.*** emphasized the buffering role of education, social cohesion, and faith-based coping in sustaining resilience during political crises [23, 31]. These convergent findings suggest that resilience operates as both a personal and collective construct, shaped by sociocultural context, identity, and institutional support.

**Table 3:** Multiple linear regression that was conducted to identify predictors of adaptive coping behaviors as measured by the brief resilient coping scale (BRCS)

Predictors	B	SE	β	t	*p*-value	95% CI for B	VIF
**Constant**	13.533	0.981	—	13.798	< 0.001	11.604–15.461	—
Gender (Female)	−0.417	0.317	−0.065	−1.316	0.189	−1.041–0.206	1.108
Age	0.662	0.155	0.216	4.259	< 0.001*	0.356–0.967	1.175
Marital Status	0.06	0.244	0.012	0.247	0.805	−0.419–0.540	1.042
Educational Level	0.411	0.18	0.109	2.28	0.023*	0.057–0.765	1.046
PHQ-9 (Depression)	0.022	0.039	0.044	0.565	0.572	−0.055–0.099	2.803
PSS (Perceived Stress)	−0.113	0.029	−0.248	−3.927	< 0.001*	−0.170 – −0.057	1.824
GAD-7 (Anxiety)	−0.015	0.043	−0.025	−0.344	0.731	−0.100–0.070	2.454

This table summarizes the results of a multiple linear regression analysis operated to illustrate the sociodemographic and psychological predictors of adaptive coping. Significant predictors included age, educational level, and perceived stress (PSS), while gender, marital status, depression (PHQ-9), and anxiety (GAD-7) were not significant contributors. Model statistics indicate a moderate fit with R² = 0.157

A *p*-value < 0.05 was considered significant

Taken together, the evidence indicates that non-displaced civilians exposed to regional conflict through media and cultural affiliation experience a dual burden of heightened anxiety and eroded coping. Strengthening community-level resilience and fostering adaptive coping frameworks—particularly for youth and socioeconomically vulnerable groups—should therefore be central to mental-health strategies in conflict-adjacent settings.

A considerable proportion of participants perceived that regional political conflict disrupted their daily functioning, particularly their ability to work, study, and manage household responsibilities. This sense of functional impairment was most prominent among younger, unmarried, and less-educated individuals—groups that also reported heightened emotional distress. These patterns suggest that indirect exposure to conflict can erode not only emotional well-being but also perceived capacity to perform social and occupational roles, reflecting the behavioral dimensions of vicarious trauma.

Consistent with prior regional evidence, ***Hendawy et al.*** and ***Najem et al.*** identified media exposure and emotional identification with conflict zones as key contributors to psychological distress and functional disruption among neighboring civilian populations [24, 33]. Najem et al. further showed that fear of war affected mental health indirectly through intolerance of uncertainty—a mechanism likely relevant to our findings [24]. Other regional studies similarly describe widespread disruption of education, employment, and family stability as downstream effects of prolonged violence, often accompanied by collective trauma and emotional numbing [23, 34].

Internationally, research from Kashmir, Syria, and South Asia supports these observations, showing that persistent political instability reduces individuals’ capacity to function and adapt, particularly in populations with low socioeconomic resilience and limited institutional support [30, 31, 35]. ***Barron and Abdallah***, and ***Canetti et al.***, emphasize that perceived threat and political oppression predict not only poorer mental health but also greater functional impairment and diminished community participation [22, 32]. Collectively, this evidence highlights how indirect conflict exposure—through shared identity, continuous media engagement, and geopolitical proximity—can profoundly affect psychological and functional health in non-combatant populations such as those in Egypt.

This study emphasizes the often-overlooked psychological burden among non-displaced civilians in conflict-neighboring regions, revealing how indirect exposure (through media and emotional sympathy) can substantially affect mental health. These findings call for regional mental health approaches that address vicarious trauma, including primary care screening, community-based support, and mental health awareness campaigns, especially for youth and active social media consumers.

**Table 4:** Association between perceived functional impact of political conflict and mental health indicators and coping behavior (N = 394)

Perceived conflict impact	PHQ-9		PSS		BRCS		GAD-7	
	Mean ± SD	95% CI	Mean ± SD	95% CI	Mean ± SD	95% CI	Mean ± SD	95% CI
No difficulty; (n = 116)	6.97 ± 5.54^b, c,d^	5.95–7.98	17.86 ± 7.03^b, c,d^	16.57–19.16	14.06 ± 3.55^b, c,d^	6.46–8.32	7.39 ± 5.06^b, c,d^	13.41–14.71
Somewhat difficult; (n = 217)	9.8 ± 5.92^a, c,d^	9.00–10.59	21.54 ± 6.57^a, c,d^	20.66–22.42	13.07 ± 3.04^a^	9.52–10.90	10.21 ± 5.14^a, d^	12.66–13.48
Very difficult; (n = 40)	14.35 ± 5.70^a, b^	12.53–16.17	25.68 ± 4.60^a, b^	24.20–27.15	13.25 ± 2.63^a^	9.45–12.40	10.93 ± 4.63^a, d^	12.41–14.09
Extremely difficult; (n = 21)	16.48 ± 6.35^a, b^	13.59–19.36	27.1 ± 4.52^a, b^	25.04–29.15	14.24 ± 3.02^a^	14.00–17.90	15.95 ± 4.28^a, b,c^	12.87–15.61
Overall p-value	< 0.001*		< 0.001*		0.031*		< 0.001*	
(η²)	(0.170, L)		(0.149, L)		(0.137, M–L)		(0.022, S–M)	

This table illustrates the mean scores of depressions (PHQ-9), perceived stress (PSS), anxiety (GAD-7), and coping behavior (BRCS) across different levels of perceived functional impact from political conflict, analyzed by one-way ANOVA with LSD post-hoc comparisons. The 95% confidence interval (CI) indicates the range where the true population mean is expected to fall with 95% confidence; non-overlapping intervals suggest meaningful group differences. Effect size (η²) reflects the proportion of variance explained and is interpreted as small (0.01–0.05), moderate (0.06–0.13), or large (≥ 0.14). Labels (S–M), (M), (M–L), and (L) indicate the relative magnitude of effect. Superscripts (a, b, c, d) indicate significant pairwise comparisons between categories

^a^: statistically significant from No difficulty category at p-value ≤ 0.05

^b^: statistically significant from Somewhat difficult category at p-value ≤ 0.05

^c^: statistically significant from Very difficult category at p-value ≤ 0.05

^d^: statistically significant from Extremely difficult category at p-value ≤ 0.05

## Conclusion

There is significant psychological burden experienced by Egyptian citizens indirectly exposed to Middle Eastern political conflict, particularly the 2023 Gaza war. High levels of depression, anxiety, and perceived stress were reported, especially among younger, female, unmarried, and lower-educated individuals. Frequent exposure to war-related media and personal connections to conflict zones emerged as key predictors of distress. Moreover, low resilient coping scores underscore the urgent need for targeted mental health interventions. These findings call for proactive public health strategies to strengthen psychological resilience and address the silent toll of regional instability on non-displaced populations.

### Study limitations and strengths

This study’s cross-sectional design precludes causal inference, and the single-site sample from Ismailia Governorate may limit generalizability. Ismailia’s border location along the Suez Canal and its history of displacement and resettlement during past wars may influence psychological responses differently from other Egyptian regions. Furthermore, cultural, economic, and demographic heterogeneity across Egypt—including urban versus rural settings and Upper versus Lower Egypt—further restricts extrapolation of the findings.

The exclusion of individuals with chronic medical or psychiatric conditions, as well as members of the armed forces and police, was intended to minimize confounding from pre-existing illness and occupational stressors; however, this may have reduced prevalence estimates by excluding groups with higher baseline vulnerability, while the healthcare-based sampling could conversely overrepresent individuals experiencing psychosomatic or stress-related complaints.

Self-reported data are subject to recall and social desirability bias. Indirect conflict exposure was assessed using a single, unvalidated item designed to capture perceived functional disruption; while justified for exploratory use, this measure requires future validation to establish reliability and convergent validity with related constructs such as media exposure or personal ties to conflict zones. Additionally, cultural stigma surrounding mental illness may have led to underreporting of symptoms. Finally, media exposure and personal connections were not quantitatively measured, which limits exploration of indirect exposure pathways.

Despite these limitations, the study holds several strengths. It is among the few to investigate the psychological and functional effects of regional political conflict among non-displaced civilians in the Middle East, using validated instruments (PHQ-9, GAD-7, PSS-10, BRCS) within a well-defined community sample. The focus on a border governorate with historical conflict exposure adds contextual depth and relevance, offering valuable insight into the indirect mental health burden of ongoing regional instability.

### Recommendations

This study recommends integrating mental health screening into primary care, promoting resilience-building programs for vulnerable groups, and encouraging mindful media consumption. Educational institutions should foster mental health awareness, while policymakers must invest in mental health infrastructure. Future research should assess long-term outcomes and the impact of community-based interventions.

## Data Availability

Data that supports the findings of this article are available through the corresponding author upon reasonable request.
